# External Ophthalmomyiasis Caused by a Rare Infesting Larva, *Sarcophaga argyrostoma*

**DOI:** 10.1155/2013/850865

**Published:** 2013-12-22

**Authors:** Shmuel Graffi, Avi Peretz, Amos Wilamowski, Heather Schnur, Fouad Akad, Modi Naftali

**Affiliations:** ^1^Department of Ophthalmology, Baruch Padeh Medical Center Affiliate of the Faculty of Medicine, Bar-Ilan University, 15208 Poriya, Israel; ^2^Clinical Microbiology Laboratory, Baruch Padeh Medical Center Affiliate of the Faculty of Medicine, Bar-Ilan University, 15208 Poriya, Israel; ^3^Laboratory of Entomology, Ministry of Health, 91342 Jerusalem, Israel

## Abstract

*Purpose*. External ophthalmomyiasis (EO) is caused by infesting larvae belonging to various species of flies. Most documented cases result from sheep (*Oestrus ovis*) and Russian (*Rhinoestrus purpureus*) botfly larvae, but we recently discovered a rare case of EO caused by flesh fly (*Sarcophaga argyrostoma*) larvae. Here, we report the case of a patient with EO who had been hospitalized and sedated for 1 week because of unrelated pneumonia. *Methods*. Case report. *Results*. A total of 32 larvae were removed from the adnexae of both eyes. Larvae identification was confirmed through DNA analysis. Treatment with topical tobramycin resulted in complete resolution of EO. *Conclusion*. EO can be caused by *S. argyrostoma*, and the elderly and debilitated may require extra ocular protection against flies during sedation.

## 1. Introduction

The term ophthalmomyiasis implies the presence of fly larvae within (internal ophthalmomyiasis) or around (external ophthalmomyiasis; EO) the orbital region. EO is the more benign infestation and is commonly caused by sheep (*Oestrus ovis*) [1] or Russian gad (*Rhinoestrus purpureus*) flies botflies. Although various flies may cause ophthalmomyiasis [2], no previous reports of EO resulting from *Sarcophaga argyrostoma* (flesh fly), could be found in the literature (Google Scholar, PubMed search terms: “Ophthalmomyiasis and *S. argyrostoma*”). This gray-coloured fly belongs to the Sarcophagidae family and exists worldwide, usually around corpses [3] and, to a much lesser extent, in open wounds. Here, we report a recent case of hospital-acquired EO resulting from *S. argyrostoma* larvae.

## 2. Case Report

A 91-year-old man from Northern Israel, where agriculture is abundant, was admitted to a local hospital for pneumonia and was placed in the care of the Department of Internal Medicine. Because of a general deterioration in health, the man was sedated and intubated. One week later, in the late afternoon of October 28, 2013, a nurse discovered larvae on the palpebral fissure of both eyes.

**Figure 1:**
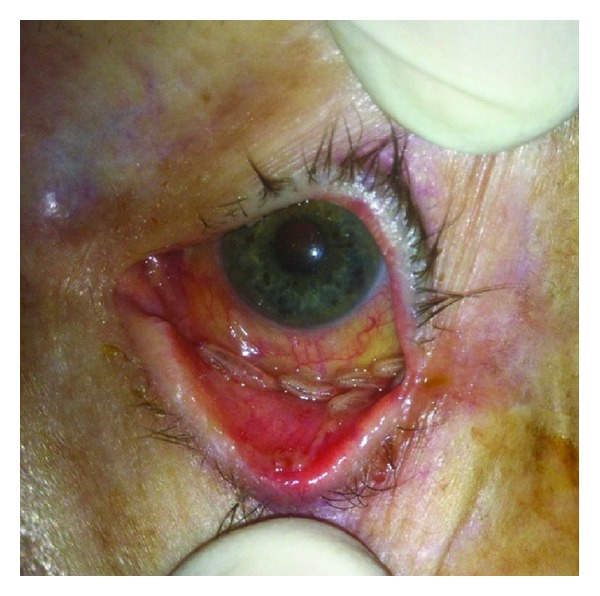
Retraction of the left eyelid revealed multiple larvae resting in the inferior fornix. Mucopurulent discharge can also be seen.

A bedside ophthalmic examination revealed multiple motile larvae in the inferior fornices of both eyes (Figure 1). Retraction of the lids enabled visualization of most of the larvae that were avoiding the light. The conjunctiva was diffusely congested, and a mucopurulent secretion was present in the inferior fornices. Other findings of the examination were unremarkable.

Topical oxybuprocaine hydrochloride (0.4%) drops were administered in both eyes as a topical anaesthetic. A total of 13 and 19 larvae were easily removed from the right and left eyes, respectively. All larvae measured 7-8 mm in length and were preserved in 70% alcohol before being sent to the national entomology laboratory for identification. A secretion specimen was also collected for culture.

The patient was treated with topical tobramycin ophthalmic ointment 3 times a day. After 1 week, the conjunctiva was no longer congested, completely regressing back to its preinflammatory state. All specimens were culture negative, demonstrating no bacterial growth.

Preserved larvae were inspected by an experienced entomologist and based on their morphology were found to be 3-4 days old of *Sarcophaga* sp. in their second stage of development. Because accurate larvae identification is more difficult in the early developmental stages, a genomic DNA was extracted from the larva and subjected to polymerase chain reaction (PCR) to amplify a segment from the cytochrome oxidase gene subunit I (COI); the sequence of the amplified segment revealed 97% similarity to COI gene from *Sarcophaga argyrostoma* (Voucher NICC0091, gb: JQ413450.1). On the basis of the above findings, identification of the larvae was confirmed.

## 3. Discussion

EO caused by *S. argyrostoma* can occur in hospitals and nursing homes. This report describes a rare case of a hospital-acquired EO caused by *S. argyrostoma.* Identification was confirmed by DNA analysis of the second-stage larvae in a molecular biology laboratory. In our case, DNA analysis was used because it is almost impossible to morphologically identify larvae in the second stage of development. An identification method for third-stage larvae involves examining the development of the posterior spiracular disc; however, this method is only moderately reliable. Once larvae reach adulthood, identification can be based purely on morphology. Unfortunately, in our case, all larvae were instantly killed at the time of extraction upon alcohol immersion.

A thorough literature search on PubMed and Google Scholar (search terms: “external ophthalmomyiasis” and “*Sarcophaga*”) found no previous reports regarding this source of infestation. One case of EO caused by *Sarcophaga crassipalpis *had a similar presentation as described here [4], but no photos of the infection were included in the publication. In that study, *S. crassipalpis* larvae removed from the eye were reared to adulthood for identification.

In the setting of routine hospital care, larvae were found maturing in a patient's eye at a relatively advanced stage of development. Previous cases of hospital-acquired myiasis have been reported [5]. Given that many hospitalized patients are elderly and/or debilitated, both of which increase the likelihood of myiasis [6], the presence of flies in a hospital should not be tolerated.
